# Metabolic Phenotyping of Adipose-Derived Stem Cells Reveals a Unique Signature and Intrinsic Differences between Fat Pads

**DOI:** 10.1155/2019/9323864

**Published:** 2019-05-14

**Authors:** Camille Lefevre, Baptiste Panthu, Danielle Naville, Sylvie Guibert, Claudie Pinteur, Bénédicte Elena-Herrmann, Hubert Vidal, Gilles J. P. Rautureau, Anne Mey

**Affiliations:** ^1^Univ Lyon, CarMeN Laboratory, INSERM, INRA, INSA Lyon, Université Claude Bernard Lyon 1, 69921 Oullins Cedex, France; ^2^Hospices Civils de Lyon, Faculté de Médecine, Hôpital Lyon Sud, 69921 Oullins Cedex, France; ^3^Univ Lyon, CNRS, Université Claude Bernard Lyon 1, Ens de Lyon, Institut des Sciences Analytiques, UMR 5280, 5 rue de la Doua, F-69100 Villeurbanne, France; ^4^Institute for Advanced Biosciences, CNRS UMR 5309, INSERM U1209, Université Grenoble Alpes, Grenoble, France

## Abstract

White adipose tissues are functionally heterogeneous and differently manage the excess of energy supply. While the expansion of subcutaneous adipose tissues (SAT) is protective in obesity, that of visceral adipose tissues (VAT) correlates with the emergence of metabolic diseases. Maintained in fat pads throughout life, adipose stem cells (ASC) are mesenchymal-like stem cells with adipogenesis and multipotent differentiation potential. ASC from distinct fat pads have long been reported to present distinct proliferation and differentiation potentials that are maintained in culture, yet the origins of these intrinsic differences are still unknown. Metabolism is central to stem cell fate decision in line with environmental changes. In this study, we performed high-resolution nuclear magnetic resonance (NMR) metabolomic analyses of ASC culture supernatants in order to characterize their metabolic phenotype in culture. We identified and quantified 29 ASC exometabolites and evaluated their consumption or secretion over 72 h of cell culture. Both ASC used glycolysis and mitochondrial metabolism, as evidenced by the high secretions of lactate and citrate, respectively, but V-ASC mostly used glycolysis. By varying the composition of the cell culture medium, we showed that glutaminolysis, rather than glycolysis, supported the secretion of pyruvate, alanine, and citrate, evidencing a peculiar metabolism in ASC cells. The comparison of the two types of ASC in glutamine-free culture conditions also revealed the role of glutaminolysis in the limitation of pyruvate routing towards the lactate synthesis, in S-ASC but not in V-ASC. Altogether, our results suggest a difference between depots in the capacity of ASC mitochondria to assimilate pyruvate, with probable consequences on their differentiation potential in pathways requiring an increased mitochondrial activity. These results highlight a pivotal role of metabolic mechanisms in the discrimination between ASC and provide new perspectives in the understanding of their functional differences.

## 1. Introduction

White adipose tissue is an interesting source of multipotent stem cells sharing properties with mesenchymal stem cells and used for clinical applications. Indeed, in addition to their differentiation potential, adipose stem cells (ASC) display stromal functions (i) by supporting the growth of other stem cells [1], (ii) by controlling local inflammation through the secretion of cytokines or by the interaction with immune cells [2], and (iii) by controlling energy metabolism pathways by the secretion of hormones such as adiponectin [3]. For a long time, ASC properties have been used in regenerative medicine and for cell therapy [4] with an increasing interest for the use of their secretome [5]. However, their contribution to adipose tissue homeostasis and expansion in obesity is not clear, notably due to the functional heterogeneity of adipose fat pads.

White adipose tissue is split into different body regions with two main areas, the subcutaneous (SAT) and the visceral (VAT) white adipose depots that play distinct roles in the control of energy metabolism. Indeed, SAT expansion is protective in obesity while VAT expansion promotes the metabolic complications of obesity such as resistance to insulin and type 2 diabetes [6].

It has been reported that SAT and VAT have distinct functional properties regarding their capacity of fatty acid storage and the control of inflammation [7]. The metabolic protection by SAT is attributed to its ability to trap free fatty acids through triglyceride esterification (lipogenesis) protecting other organs from lipotoxicity [8] while the deleterious effect of expanding VAT is attributed to its higher lipolytic activity favoring the release of free fatty acids [6] and the delivery of proinflammatory cytokines such as IL6 [9]. It has been shown that VAT expansion occurs when the storage capacity of SAT is saturated [10], a process associated with stem cell proliferation and differentiation [11]. Whether this stem cell mobilization, to produce new adipocytes in obesity, is restricted to VAT remains controversial [12, 13], but it reveals distinct metabolic dialogs between stem cells and their environment.

Reinforcing the differences between adipose tissues, functional differences exist between ASC from distinct depots, with ASC from SAT (S-ASC) showing higher abilities to proliferate [14], to survive [7, 15], to accumulate lipids [7, 14], and to differentiate into adipocytes [16–18] than ASC from VAT (V-ASC). Interestingly, these differences observed with isolated ASC cultivated *in vitro* are thus intrinsic indicating distinct fate for ASC in distinct adipose depots [14].

Metabolic adaptation is central to the balance between proliferation and differentiation of stem cells that support tissue homeostasis and adaptation. It is assumed that proliferating stem cells exhibit a glycolytic metabolic program supporting cell synthesis [19] while the switch to mitochondrial activity and oxidative phosphorylation is required for differentiation [20]. In multipotent mesenchymal stem cells that can give rise to chondrocytes, osteoblasts, and adipocytes, the switch between glycolysis and oxidative phosphorylation further directs the lineage decision between osteogenesis and chondrogenesis according to the requirements of the mature cell phenotype for oxygen supply [21]. Similarly, ASC, which are intended to generate adipocytes in white adipose tissues, may preferentially depend on metabolic pathways related to fatty acid synthesis.

In this article, to further understand the intrinsic differences between ASC from distinct adipose depots, we addressed their metabolic features. To reach this goal, we have set up efficient isolation and culture conditions of ASC allowing the amplification of stem cells from SAT and from VAT collected from mice. The metabolic pathways involved in the maintenance of ASC were defined by using proton nuclear magnetic resonance (^1^H-NMR). By quantifying the metabolites in the extracellular culture medium, we measured the consumption and secretion rates of a variety of metabolites to characterize and to compare the metabolic profiles during cell cultures of ASC from distinct adipose depot. Our results indicate that V- and S-ASC present significant metabolic differences among which the role of glutaminolysis is noticeable. Our data also shed new light on the balance between glycolysis and mitochondrial activity of ASC under the influence of the depot of origin.

## 2. Materials and Methods

### 2.1. Isolation and Culture of ASC

Mouse studies were performed with the approval of the Regional Committee of Ethics for Animal Experiments. After one week acclimatization, subcutaneous and epididymal adipose tissues were harvested from 10-12-week-old male C57BL/6J mice (Envigo, Gannat, France) killed by cervical dislocation. Adipose tissues were removed and left on ice in Hanks' balanced salt solution (Dutscher, Brumath, France) supplemented with 100 U/ml penicillin and 100 *μ*g/ml streptomycin (Gibco, France) until isolation of adipose-derived stem cells. The adipose tissues were successively washed with Hanks solution and Dulbecco's phosphate balanced solution (PAA, France) before being torn into pieces. Pieces were incubated for 1 hour at 37°C in digestion buffer containing DPBS with calcium and magnesium supplemented with 2 mM glucose (Sigma-Aldrich, France), 1% bovine serum albumin (Dominique Dutscher), and 1 mg/ml collagenase (Sigma-Aldrich, France) and vortexed every 10 minutes. At the end of the process, digested adipose tissues were filtered on a 100 *μ*m nylon filter (Dutscher) and suspended in culture medium containing Dulbecco's modified Eagle's medium-high glucose (DMEM-HG) supplemented with 2 mM L-glutamine-L-alanyl (stable glutamine), 1 mM pyruvate, 100 U/ml penicillin, 100 *μ*g/ml streptomycin, 25 mM hepes, and 10% fetal calf serum from South America (all culture medium components were from Dominique Dutscher, France). The cells were centrifuged for 5 minutes at 600 g at room temperature. The pelleted stromal fractions were suspended in culture medium supplemented with 100 *μ*M ascorbic acid (Sigma-Aldrich, France), 5 ng/ml human bFGF (eBioscience, France), and 5 U/ml heparin sodium (Sigma-Aldrich) and plated in 2 wells of 9.6 cm^2^ (Dutscher). At confluency, cells were dissociated by trypsinization (0.05% trypsin-EDTA) and plated at a density of 1 × 10^4^ cells by cm^2^. This was the first passage. All the analyses were performed with cells collected after the 2^nd^ passage. For cumulative growth curves, all the passages were performed like the first one.

Cells were cultured in a humidified 5% CO_2_ atmosphere at 37°C, and medium was changed every two or three days except for metabolomic experiments.

### 2.2. Flow Cytometry

The cultured ASC were retrieved at passage 2 by trypsin digestion. After washing in PBS, cells were first incubated with the fixable viability dye eFluor 506 (Life Technologies SAS, France) in PBS. Cells were next suspended in fluorescence-activated cell sorting (FACS) buffer (10% BSA (fraction V, Euromedex), 0.1% NaN_3_ in PBS) supplemented with the mixture of cell surface marker antibodies or their isotype controls. Antibodies used were CD45 APC-eFluor 780, CD31 PE-Cy7, CD90.2-FITC, and CD29-PE all from eBioscience (Thermo Fisher, France) and PdgfR*α*- (CD140a-) BV421 and Sca-1 BUV395 from BD Biosciences (France). After 30-minute incubation on ice, cells were washed in FACS buffer and fixed in 3.7% formaldehyde. Acquisitions were performed using the facilities of the technical platform AniRA of the SFR Biosciences Gerland-Lyon Sud (US8/UMS3444) with an LSRII flow cytometer (BD Biosciences) equipped with 355, 488, and 633 nm lasers. Analyses were performed using the cloud-based platform Cytobank (http://www.cytobank.org).

### 2.3. Adipogenic Differentiation

For adipocyte differentiation, ASC were amplified until 80% of confluency was reached and culture medium replaced by differentiation medium containing Dulbecco's modified Eagle medium/Nutrient Mixture F12 (DMEM/F12; Dominique Dutscher, France), FBS 10% (Hyclone, France), 100 U/ml penicillin, 100 *μ*g/ml streptomycin, 2 mM glutamine, 1 *μ*g/ml insulin, 0.5 *μ*M dexamethasone, 2 nM Triiodo-L-Thyronine 3 (T3), 0.5 *μ*M 3-isobuthyl-1-methylxanthine (IBMX), 2 *μ*M rosiglitazone, and 10 *μ*g/ml transferrin, all from Sigma-Aldrich. After 7 days, adipocyte differentiation was measured by Oil red O staining of the neutral triglyceride and lipids. Cells were fixed in 10% formaldehyde (Carlo Erba, France) for 1 hour then washed with 60% isopropanol (Carlo Erba) twice, dried and colored by incubation for 10 minutes with 0.2% Oil red O (Sigma-Aldrich) in isopropanol. Brightfield images were taken with an optic microscope with a ×20 magnification. The expression of adipogenic genes (*Pparγ*, *Dgat2*, *and Hsl*) was measured by RT-qPCR.

### 2.4. Chondrogenic Differentiation

ASC were induced to differentiate into chondrocytes using the completed StemXVivo Chondrogenic differentiation medium (R&D Systems, France). Briefly, 2.5 × 10^5^ cells were washed once in StemXVivo Chondrogenic base medium, resuspended in 0.5 ml of the StemXVivo Chondrogenic differentiation medium, and incubated in 15 ml falcon at 37°C and 5% CO_2_. A pellet of 1-2 mm was formed. The chondrogenic differentiation medium was changed every 2-3 days. The pellet was harvested after 21 days of differentiation and fixed in 4% formaldehyde for 4 h at 4°C. The fixation was stopped with glycine 1 M (Sigma-Aldrich), and the pellet was washed with PBS before being incorporated in CryoFix Gel (BioGnost, France), frozen at -80°C, and then cut with a microtome (CryoStar NX50, Thermo Scientific). The pellet was stained for 30 minutes with alcian blue 8GX 1% (BioGnost) dye which colors glycosaminoglycans in cartilage. The nucleus was stained for 1 minute with a 0.1% fast red solution (Sigma-Aldrich), and the section was included in pertex. Images of 10 distinct fields by sample were obtained with an optic microscope (20x objective), and analysis was performed using the ImageJ software application Fiji to quantify the ratio of the alcian blue color surface to the total cell surface.

### 2.5. Osteogenic Differentiation

ASC were induced into osteocytes using the StemXVivo Mouse/Rat osteogenic/adipogenic supplement (R&D Systems). Briefly, 7.6 × 10^4^ cells were suspended in 1.5 ml of complete medium and plated in a 24-well dish. After 1 day, cells were at 60-70% confluency. Cells were purged 3 h in StemXVivo osteogenic/adipogenic base media. Osteogenic differentiation was induced with 1 ml of StemXVivo Osteogenic differentiation media and was changed every 3-4 days. After 21 days, the cells were fixed for 15 minutes using 10% formaldehyde in PBS at room temperature. The osteogenic differentiation was revealed using 40 mM alizarin red dye (which stains calcium deposition) pH 4.3 (Sigma-Aldrich) for 20 minutes at room temperature. Brightfield images were taken with an optic microscope with a ×20 magnification. The expression of the osteocyte-specific genes *Dmp1* and *Gdf15* was measured by RT-qPCR.

### 2.6. Real-Time Quantitative PCR (RT-qPCR)

The RNeasy Mini Kit (Qiagen, France) was used to extract RNA, following the provider instructions, from ASC that had been differentiated or not. The reverse transcription was performed using the Takara reverse transcriptase kit (Ozyme, France). RT-qPCR was performed using the TaqMan fast advanced master mix (Biosystems, France). Samples were run in duplicate. Gene expression levels were calculated using the Rotor-Gene Q series software and normalized using the mouse 40S ribosomal protein S17 (*Rs17*) as the housekeeping gene. The primers used are listed in Table 1.

### 2.7. Protein Analysis and Western Blot

ASC were cultivated until confluency and rinsed with PBS after removal of the culture supernatant. Whole cell lysates were prepared by adding per well of 9.6 cm^2^, 300 *μ*l of lysis buffer containing 1% IGPAL, 0.5% sodium deoxycholate, 0.1% sodium dodecyl sulfate (SDS), PBS without calcium without magnesium, 5 mM EDTA, 1 mM NaVO_4_, 20 mM NAF, and 1 mM DL-dithiothreitol, and supplemented with a protease Inhibitor Cocktail (all reagents were obtained from Sigma, France). After incubation of 30 min on ice, cell lysates were centrifuged at 12,000 g for 20 min at 4°C to remove insoluble fragments. The total protein content in the supernatant was determined using the Pierce BCA Protein Assay Kit (Thermo Scientific, France) and BSA as the standard curve. For western blot analysis, proteins were denatured with loading buffer (25 mM Tris HCl, 6% glycerol, 0.5% SDS, 2% *β*-mercaptomethanol, 0.005% bromophenol blue) at 75°C for 10 min, and 20 *μ*g protein per well was loaded. Proteins were separated on a 12% SDS-polyacrylamide gel and transferred on a PVDF membrane. The membranes were blocked in saturation buffer (0.3% Tween, 5% low-fat milk in TBS) for 2 h at room temperature and next incubated overnight at 4°C with the primary antibody diluted in saturation buffer. The following primary antibodies were used: rabbit anti-UCP2 (1/500, BioLegend, France) and mouse anti-*α*-tubulin (1/2000, Sigma, France). After washing three times with TBS-0.3% Tween, the membrane was incubated with the HRP-conjugated anti-rabbit antibody (Bio-Rad, France). After washing, peroxidase activity was detected by chemiluminescence using the Luminata Classico western HRP substrate (Millipore, France). Detection was made using the ChemiDoc XRS+ imaging system (Bio-Rad), and analysis was performed using the Quantity One software (Bio-Rad). Data were normalized relatively to *α*-tubulin.

### 2.8. Statistical Analysis

All results are expressed as means ± SEM. Student's *t*-test was used to evaluate the probability of significant differences between the ASC samples isolated from distinct fat pads. One-way ANOVA followed by Tukey's multiple comparison test was used when more than two conditions were compared. A *p* value < 0.05 was considered as significant. GraphPad Prism 5.0 software was used for all statistical analyses.

### 2.9. Metabolomic Analyses

#### 2.9.1. Exometabolome Sample Preparation

Metabolite concentrations were measured in supernatants of ASC placed for the indicated time in culture medium composed of DMEM without glucose, without pyruvate, without glutamine, and without red phenol (PAN-Biotech, France) supplemented with 25 mM glucose, antibiotics, 20% FCS, 25 mM hepes, nonessential amino acid 1x (PAA, France), 4 mM L-glutamine (Corning, France), 1 mM pyruvate (Dutscher, France), 100 *μ*M ascorbic acid, 5 ng/ml human bFGF, and 5 U/ml heparin sodium. This medium had the same nutrient composition as the growth culture medium and only differed by the absence of phenol red, the replacement of L-glutamine-L-alanyl (2 mM) by L-glutamine (4 mM). Cells were seeded at a density of 2 × 10^4^ cells per cm^2^ in a 12-well dish and cultured in 1.5 ml of culture medium. After 24 h, as the cells reached 70 to 80 percent of confluency, the medium was replaced by 500 *μ*l of fresh culture medium, and the culture was maintained for 24, 48, or 72 h, as indicated in the figure legends. Culture medium in wells without cells was processed in the same ways to obtain the initial concentrations of metabolites in the medium (control). For studies examining the role of pyruvate, glucose, and glutamine supplies, cells were seeded as previously indicated and cultivated for 72 h. At confluency, culture medium was replaced by culture medium without pyruvate nor hepes and supplemented with the indicated concentrations of glucose and glutamine. The other components were at the concentrations indicated above. At the end of the culture, supernatants were collected and snap-frozen at -20°C for NMR analyses. For the preparation of exometabolome samples, 200 *μ*l of centrifuged cell culture supernatant was supplemented with 400 *μ*l of phosphate buffer pH 7.4 (160 mM Na_2_HPO_4_, 30 mM NaH_2_PO_4_, 1 mM TSP, and 3 mM NaN_3_ in 100% D_2_O) [22]. Samples were analyzed in 5 mm NMR tubes containing 550 *μ*l of the sample mix. Control culture mediums were analyzed in parallel during each NMR session.

#### 2.9.2. NMR Acquisition

All NMR experiments were acquired on a 600 MHz Bruker NMR spectrometer equipped with a 5 mm TCI cryoprobe at 30.0°C. A cooled SampleJet autosampler enabled high throughput data acquisition. A standard ^1^H-1D NMR pulse sequence nuclear Overhauser effect spectroscopy (NOESY) with z-gradient and water presaturation (Bruker pulse program *noesygppr1d*) was recorded on each sample, with a total of 128 transient free induction decays (FID) and a spectral width of 20 ppm, and a relaxation delay was set to 4 seconds. The NOESY mixing time was set to 10 milliseconds, and the 90° pulse length was automatically determined for each sample (around 13 *μ*s). The total acquisition time of each sample was 12 minutes and 15 seconds.

#### 2.9.3. NMR Data Processing

All free induction decays (FIDs) were multiplied by an exponential function corresponding to a 0.3 Hz line-broadening factor prior to Fourier transform ^1^H-NMR spectra which were manually phased and referenced to the glucose doublet at 5.23 ppm using TopSpin 2.2 (Bruker GmbH, Rheinstetten, Germany). TSP was not used for data processing in this study. For multivariate analyses, residual water signal (4.85–4.67 ppm) was excluded. Spectra were divided into 0.001 ppm-wide buckets over the chemical shift range (-0.2; 9.5 ppm) using the AMIX software (Bruker GmbH).

#### 2.9.4. Spectra Analyses

Identification of the metabolites was carried out from the 1D NMR data using the software Chenomx NMR Suite 8.0 (Chenomx Inc., Edmonton, Canada) and confirmed from analysis of 2D ^1^H-^1^H TOCSY, ^1^H-^13^C HSQC, and ^1^H J-Resolved NMR spectra recorded with standard parameters. The measured chemical shifts were compared to reference shifts of pure compounds using the HMDB database [23]. Relative metabolite concentrations were determined using Chenomx software by manual fitting of the proton resonance lines for the compounds available in the database. The linewidth used in the reference database was adjusted to the width of one component of the alanine doublet. A pure standard lactate solution (1 g/l, Fisher) was used as an external concentration reference and exploited using the ERETIC2 utility from TopSpin (Bruker GmbH, Rheinstetten, Germany) to add a digitally synthesized peak to a spectrum [24]. Concentrations in the cell culture media are presented as absolute, not normalized data. They are apparent concentrations, as endogenous and FCS proteins were not removed from the NMR samples.

#### 2.9.5. Multivariate Data Analyses

Multivariate analyses were performed on NMR spectra buckets in the absence of any normalization using SIMCA-P 13 (Umetrics, Umea, Sweden) with Pareto scaled variables. Principal component analysis (PCA) was used to derive the main sources of variance within the data set, assess sample homogeneity, and exclude biological or technical outliers. Orthogonal projection to latent structure discriminant analysis (O-PLS-DA) was used to build predictive sample classification models. Results were visualized on score plots, corresponding to sample projections onto the predictive axis and the first orthogonal component of the model, and the associated loading plot. The optimal number of orthogonal components was selected using a 7-fold cross-validation procedure. The *R* ^2^ and *Q* ^2^ parameters were computed to estimate the goodness of fit and prediction, i.e., the explained and predicted variances, respectively. The O-PLS-DA models were validated using permutations under the null hypothesis (1000 times); for each permutated classification labels, *R* ^2^ and *Q* ^2^ were recalculated and compared to the original ones, and their decrease indicates the good quality of the model [25]. Metabolites involved in class discrimination were highlighted with the statistical recoupling of variables (SRV) analysis [26]. SRV corresponds to an automatic binning scheme based on the relationship of correlation and covariance between consecutive variables, which is followed by a univariate unpaired two-tailed *t*-test calculated for each variable under the Benjamini–Hochberg correction to cope with multiple testing issues [27].

## 3. Results

### 3.1. Phenotypic Analysis and Stem Cell Properties of ASC Populations

**Figure 1:**
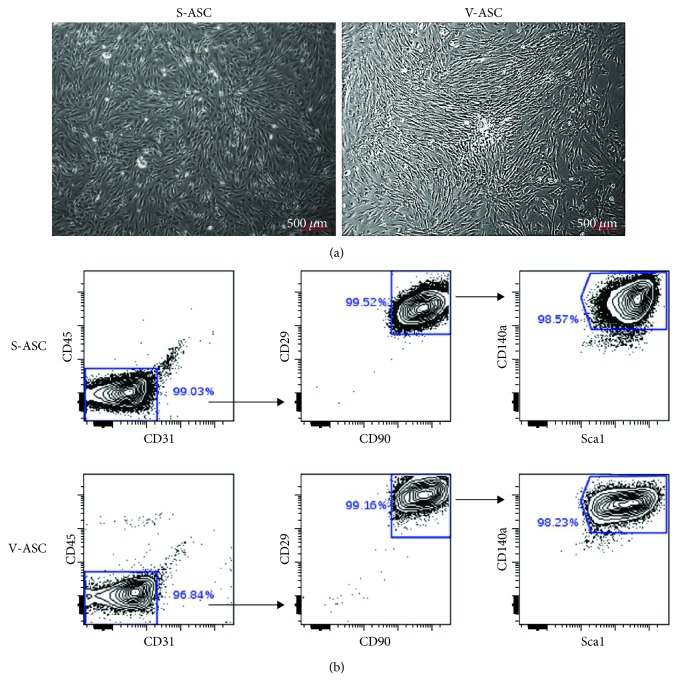
Characterization of ASC. S- and V-ASC were cultivated in ASC culture medium for 2 passages before analysis. (a) Feature of adipose-derived stem cells in culture: brightfield images of cells at confluency. (b) Membrane expression of markers of adipose stem cells CD29, CD90, Sca1, and CD140a analyzed by flow cytometry and represented as dot plot showing the percent of positive cells for the indicated markers.

**Figure 2:**
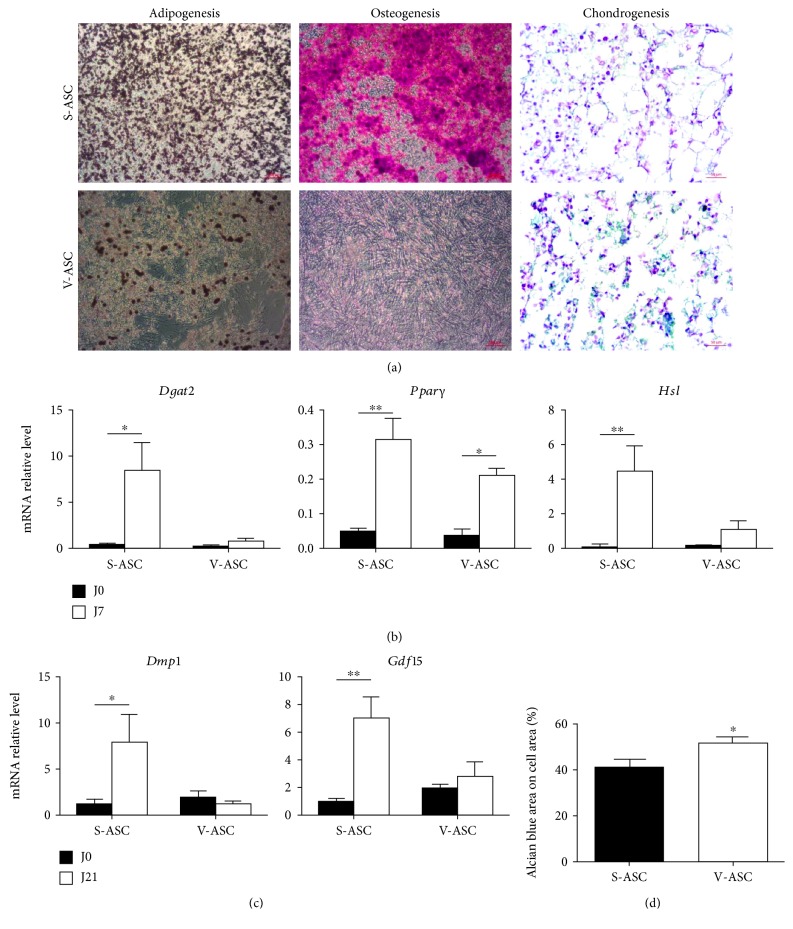
Differences between S- and V-ASC in their differentiation potential. S- and V-ASC were cultivated in ASC culture medium for 2 passages before (J0) the induction of differentiation into adipocytes for 7 days (J7) or into osteoblasts or chondrocytes for 21 (J21). (a) Brightfield images with a ×20 magnification represent adipocytes colored by Oil red O dye, osteoblasts colored with alizarin red dye, chondrocytes colored with alcian blue, and nucleus colored with fast red dye. (b) Quantification of adipogenic transcripts (*Dgat2*, *Pparγ*, and *Hsl*) by RT-qPCR analysis before and after induction of the adipogenic differentiation (*n* = 3). (c) Quantification of osteogenic genes *Dmp1* and *Gdf15*, which are, respectively, early and late markers of osteocytes, by RT-qPCR analysis before and after 21 days of osteogenic differentiation (*n* = 3). (d) Quantification of the chondrogenic differentiation by image analysis using the ImageJ software Fiji. Results present the ratio of the alcian blue-colored surface to the total cell surface and are representative of the proportion of chondrocyte-differentiated cells that are characterized by the expression of alcian blue-stained glycosaminoglycans. Analyzed pictures were from a ×20 magnification (*n* = 4). All results are mean ± SEM; statistics are from *t*-tests: ^∗^ *p* < 0.05 and ^∗∗^ *p* < 0.01.

S-ASC were isolated from subcutaneous and V-ASC from epididymal white adipose tissues in mice. ASC grew as monolayers in culture plates (Figure 1(a)). Starting from the stromal vascular fraction, our culture conditions led to the depletion in CD45+ and CD31+ cells representative of the hematopoietic and of the endothelial lineages, respectively, and to the enrichment into CD45- and CD31- cells at the end of the first passage. Analysis by flow cytometry showed that these cells were positive for mesenchymal stem cell markers (Sca1, CD29, and CD90) [28], and a fraction of them additionally expressed markers of the adipogenic potency (CD140a) (Figure 1(b)). However, these cells did not express CD24 (not shown) indicating that they were not committed to adipogenesis [29]. To confirm the mesenchymal stem cell identity of ASC, their multipotent differentiation potential was assessed [30].  Figure 2 shows that S- and V-ASC are able to differentiate into chondrocytes and into adipocytes, but only S-ASC can give rise to osteocytes, in accordance with a previous report [31]. Interestingly, both ASC had comparable efficiency to differentiate into chondrocytes, but V-ASC were less efficient than S-ASC to generate adipocytes. This was in accordance with the differences described between adipose depots regarding the fatty acid storage function. Altogether, these data confirm that our culture conditions are suitable to sustain the production of ASC from both SAT and VAT and to preserve their intrinsic functional differences.

### 3.2. Multivariate Statistical Analyses Discriminate S- and V-ASC and Highlight Differentially Secreted Metabolites

**Figure 3:**
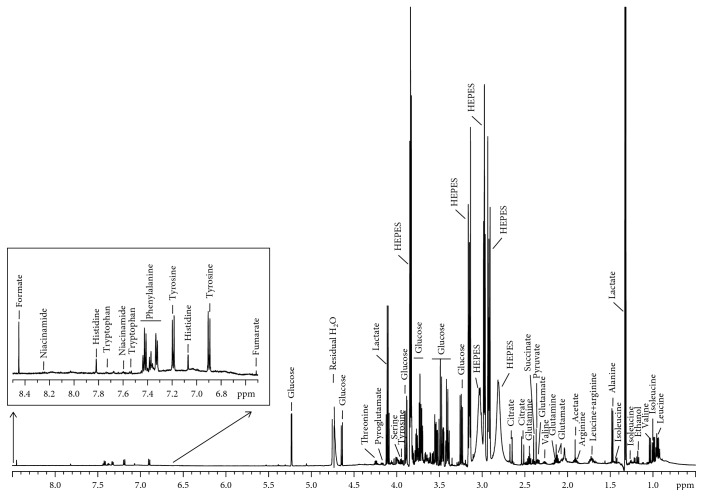
Typical ^1^H-NMR spectrum of ASC supernatant (600 MHz, 30.0°C). Spectrum from 72 h culture of S-ASC supernatant is represented. Major metabolite peak assignments are indicated.

To define the best culture conditions to analyze the exometabolome (the ensemble of metabolites in the extracellular medium), we initially performed a comparison of the extracellular metabolite concentration variations over 24 to 72 h of culture (Figure S1). Culture supernatants of ASC cultivated during 72 h were analyzed by NMR spectrometry and delivered well-resolved ^1^H-NMR metabolic profiles of the exometabolome. The ^1^H-1D spectra presented typical sharp lines corresponding to small metabolites, overlaid with broad signals from lipids or larger proteins, which appeared negligible in the case of these culture supernatants (Figure 3). At 72 h, the steady state in metabolite changes was not reached even for the most proliferative population, S-ASC, except for essential amino acids which were still not depleted in the culture medium. The steady state in amino acid consumption reached after 48 h corresponded to growth arrest. These results show that medium replacement did not influence the linear consumption or secretion of metabolites analyzed from 24 to 72 h later and that cells analyzed in the conditions described in this study were still active for the glucose and the glutamine metabolisms.

As S- and V-ASC are known to functionally differ, we conducted multivariate data analyses of their exometabolome after 72 h of culture to identify the NMR spectra regions that correlate with cell types followed by peak identification. We also used univariate metabolite concentration comparisons to complement the analysis.

Multivariate data analyses were conducted on the NMR spectra bins (0.001 ppm; 9700 NMR spectral variables) to benefit from the full spectral dynamic range of information. To get a clear view on the metabolic differences between cell types, while avoiding the complexity induced by the production or consumption status of each metabolite, the analyses were performed on the absolute value of the NMR spectra variation to the original culture medium.

**Figure 4:**
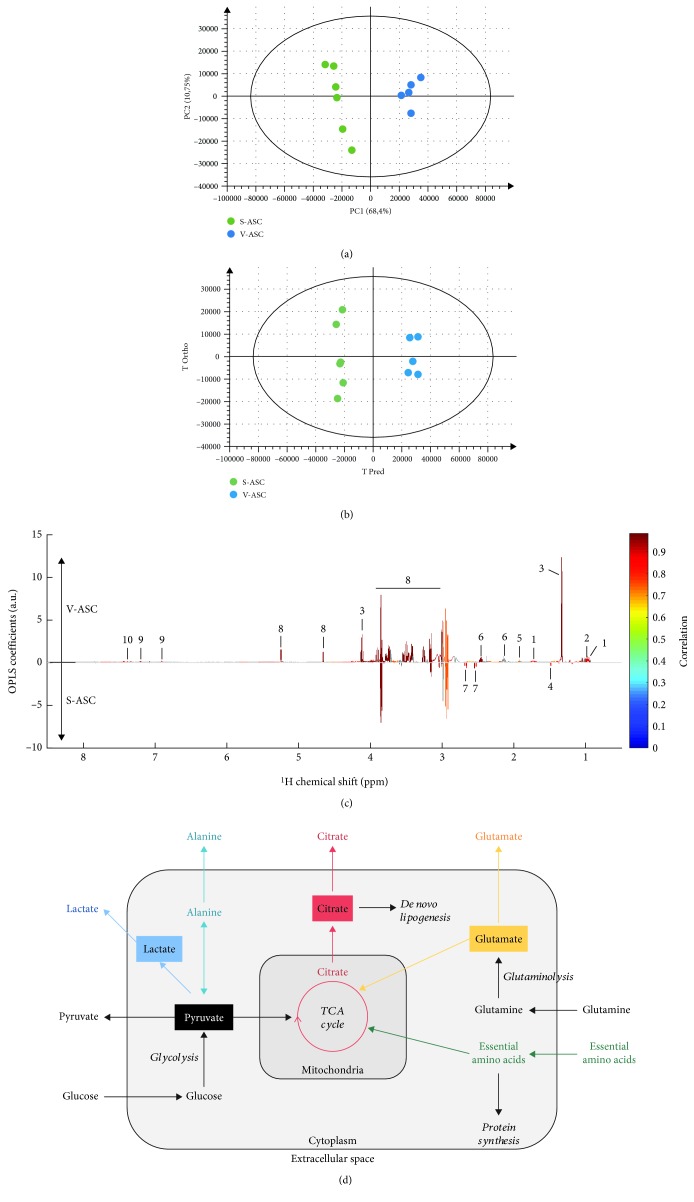
Multivariate statistical analyses of the differences between S- and V-ASC exometabolomes. High-resolution NMR multivariate data analyses unveil significant differences in extracellular metabolite variations between S-ASC and V-ASC at 75% confluency and cultivated for additional 72 h in media containing 25 mM glucose, 4 mM glutamine, 1 mM pyruvate, and nonessential amino acids as determined by NMR absolute spectra bins after subtraction of the culture medium signal analysis performed on NMR spectra buckets without normalization to the cell number. At the time of supernatant collection, cells were at confluency. (a) Untargeted principal component analysis (PCA) readily evidences the cell type as a major origin of the dataset variance. Score plot of the PCA model (PC1 and PC2) (*n* = 11, *R* ^2^ = 0.962, and *Q* ^2^ = 0.843 on 5 principal components). (b, c) Supervised multivariate data analysis (O-PLS-DA) optimizes the discrimination between both cell types after 72 h of culture. The strong discrimination of the multivariate model is shown by the high values of goodness-of-fit model parameters *R* ^2^ and *Q* ^2^ (*R* ^2^(*X*) = 0.796, *R* ^2^(*Y*) = 0.991, and *Q* ^2^ = 0.969). The discrimination robustness was validated by resampling 1000 times the model under the null hypothesis (data not shown), and the analysis of variance (CV-ANOVA) of the model led to a *p* value of 1.20 × 10^−4^. (b) Score plot of the (1 + 1) O-PLS-DA model discriminating S-ASC (in green) and V-ASC (in blue). (c) O-PLS-DA loading plot after SRV analysis and Benjamini–Hochberg multiple testing correction. The O-PLS-DA loadings reveal the influential metabolite variations on the cell type discrimination (V-ASC upper panel and S-ASC lower panel). The loading plot was complemented by color-coded correlation [26] indicating statistically significant signals. Highlighted candidate biomarkers are (1) leucine, (2) valine, (3) lactate, (4) alanine, (5) acetate, (6) glutamine, (7) citrate, (8) glucose, (9) tyrosine, and (10) phenylalanine. (d) Simplified and nonquantitative representation of the main carbon metabolic pathways in actively dividing cells. Anabolic and catabolic pathways are represented in italics in relation to the metabolite secretome. Colors identify the metabolic pathways analyzed in this study. TCA: tricarboxylic acid cycle; EAA: essential amino acids.

PCA unsupervised multivariate data analyses were first used to evaluate the dataset homogeneity and potential sample class discrimination. The dataset showed good homogeneity, though one sample from V-ASC appeared as a strong outlier on the PCA score plot and was subsequently removed from further analyses (this sample presented ethanol contamination, data not shown). A straightforward discrimination between S-ASC and V-ASC was observed on the PCA unsupervised model (Figure 4(a)). Remarkably, the first principal component of this model could explain alone 68.4% of the variance within the dataset. Those untargeted results indicate significant differences of the metabolic profile between both cell types.

To specifically target the NMR regions discriminating S-ASC and V-ASC, a supervised analysis by O-PLS-DA [32, 33] was conducted on the 9700 NMR spectral variables. We obtained a strongly discriminating O-PLS-DA model (Figure 4(b)). The analysis of NMR regions presenting the most differentially expressed peaks pointed out 10 metabolites which concentration in the culture medium was the most influenced by the presence of the distinct ASC. Glucose, leucine, valine, glutamine, tyrosine, phenylalanine, lactate, and acetate appeared to vary more in V-ASC than in S-ASC culture supernatants, while citrate and alanine varied more in S-ASC than in V-ASC culture supernatants (Figure 4(c)). These results define distinct metabolic footprints of V- and S-ASC on their microenvironment and suggest metabolic differences between both cell types.

### 3.3. Depot-Specific Features of ASC Metabolic Signatures

Careful analysis of the ^1^H-1D and 2D ^1^H-^1^H and ^1^H-^13^C NMR spectra provided the identification of 29 metabolites that were present in the S- and V-ASC supernatants after 72 h culture and that belong to a variety of biochemical classes (amino acids, sugars, and metabolic intermediates). We did not detect cell type-specific metabolites, but concentrations determined using the Chenomx software revealed quantitative differences (Table S1).

A scheme illustrating the main anabolic and catabolic pathways and key metabolites is presented in Figure 4(d). In this study, we analyzed changes at a given time in the composition of the cell culture supernatants in comparison with the cell-free culture medium placed in the same conditions. Different from the intracellular compartment, the cell culture supernatant is not a homeostatic compartment. As a consequence, the exometabolome analyzed in this study does not reflect the status of the cells at a time but rather the addition of successive changes induced by the cellular activity during culture for a given period. Therefore, the correlation between the exometabolite concentrations in the cell culture supernatants and the number of cells at a given time is not linear. That is why all the results presented in the manuscript are metabolite concentrations in the culture supernatants without any normalization and for a given period of time. However, a major attention was paid to the number of cells to minimize the contribution of this parameter to the results.

**Figure 5:**
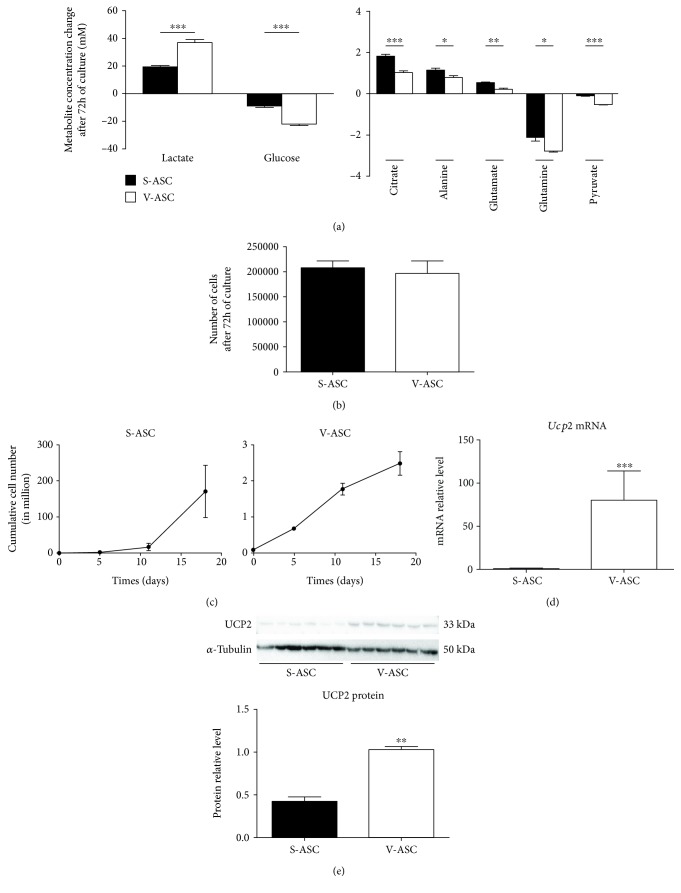
Quantitative comparison of S- and V-ASC secretomes: S- and V-ASC at 75% confluency were cultivated for 72 h in fresh culture medium containing pyruvate to reach confluency. Cells were counted as supernatants were collected for NMR analysis. Variations of the concentrations after deduction of the medium values are represented without normalization to the cell number. (a) Metabolites representing glycolysis (left panel) and other metabolites (right panel) showing significant differences between S- and V-ASC (*n* = 6). (b) Number of cells obtained after 72 h of culture with pyruvate. Cells were counted after collection of the culture supernatants (*n* = 6). (c) Cumulative growth curves: S- and V-ASC were cultivated in complete culture medium (see Materials and Methods) from the isolation to the 4^th^ passage. The number of cells after each passage is represented (*n* = 3). (d) Comparison of *Ucp2* mRNA expression in S- and V-ASC. *Ucp2* mRNA levels were quantified by RT-qPCR in S- and V-ASC harvested at the second passage. Values are normalized on the expression of the housekeeping gene *RS17* (*n* = 7). (e) Comparison of UCP2 protein content in S- and V-ASC cell lysates by western blotting. Cell lysates were from S- and V-ASC harvested at the second passage. Images (top) were obtained with the ChemiDoc XRS+ imaging system (Bio-Rad) and were analyzed (bottom) with the Quantity One software (Bio-Rad). Values were normalized relatively to the *α*-tubulin protein expression (*n* = 3). All results are mean ± SEM; statistics are from *t*-tests: ^∗^ *p* < 0.05, ^∗∗^ *p* < 0.01, and ^∗∗∗^ *p* < 0.001.

Glucose and glutamine were the most consumed substrates, and their transformation products, lactate and glutamate, were among the most secreted metabolites, along with citrate and alanine (Figures 5(a), Figure S1). When considering glycolysis, the consumption of glucose mirrored the production of lactate indicating active glycolysis in ASC. The high level of lactate secretion demonstrates a high aerobic glycolytic activity in both S- and V-ASC. This singularity was previously described as the Warburg-like effect, a type of noncancer cell metabolism associated with self-renewing stem cells [34].

On the opposite, the consumption of glutamine generated few amounts of glutamate, indicating that the carbons generated from glutaminolysis were directed elsewhere, most probably towards the production of additional metabolites and cell growth, as suggested by the strong amino acid consumptions supporting the anabolism associated with protein synthesis (Table S1). Indeed, essential amino acids (EAAs), which cannot be synthetized in mammalian cells, such as phenylalanine, histidine, and threonine, branched chain essential amino acids (BCAAs: valine, leucine, and isoleucine), and even the semi-EAA tyrosine were similarly consumed by both ASC (Table S1). The consumption of EAA is essential and inherent to proliferating cells, but the secretion rather than incorporation of glutamate and alanine (Figures 5(a)) suggests an excess of these amino acid production regarding protein synthesis in the context of stem cells.

This ensemble of observations highlights a high glycolysis associated to a Warburg-like effect and glutaminolysis activity in ASC, typical of cells in active division as confirmed by the cell count along several passages (Figure 5(c)). However, interesting differences were observed between the two ASC populations. V-ASC secreted higher lactate concentrations and S-ASC higher citrate concentrations in culture supernatants (Figures 5(a)).

Illustrating the mitochondrial activity, citrate is a metabolite produced by the tricarboxylic acid (TCA) cycle. The citrate produced by the TCA pathway inside the mitochondria can be further processed as a substrate for ATP production or partly exported to the cytoplasm to fuel the de novo lipid biosynthesis pathway (Figure 3(d)), a feature required to the neosynthesis of membranes of highly proliferating cells [35]. These intracellular metabolite fluctuations are beyond the scope of this study.

Both lactate and citrate are features of highly proliferating stem cells and are, respectively, representative of the use of pyruvate in glycolysis and in the mitochondrial TCA cycle. A high level of glycolysis is the signature of undifferentiated cells [36] while citrate can be used for *de novo* lipogenesis to support the synthesis of new cell membranes [35]. The comparison of S- and V-ASC growth curves shows that V-ASC proliferated slower than S-ASC and generated at the third passage about 10-fold less cells (Figure 5(c)) despite a higher glycolytic activity. After the third passage, S-ASC entered the exponential growth phase while V-ASC proliferation started to decline (Figure 5(c)). In this study, cells were used at passage 2 where the cell number in both populations was still quite similar as shown in Figure 5(b) ruling out the influence of the number of cells in the lower citrate secretion by V-ASC. Therefore, the difference in the balance between lactate and citrate secretions is a key feature of the difference between S- and V-ASC that suggests distinct uses of pyruvate between the two populations.

Pyruvate diversion away from the mitochondria is an active process differentiating cancer and noncancer stem cells [37]. UCP2 has been evidenced as a gatekeeper of pyruvate entry into the mitochondria that limits mitochondrial catabolism of pyruvate [38] and promotes oxidation of alternative substrates such as glutamine and fatty acids [39]. Preventing OXPHOS activation, UCP2 expressed in embryonic stem cells favors their maintenance and is repressed as pluripotency is lost [40]. Measurements of *Ucp2* transcripts (Figure 5(d)) and UCP2 protein (Figures 5(d)) in ASC revealed the higher expression in V-ASC than in S-ASC, which is consistent in V-ASC cells with a reduced pyruvate routing into the mitochondria, an elevated lactate secretion, and a majored role of glycolysis for ATP production.

Altogether, ASC present the characteristics of proliferating stem cells, with a Warburg-like effect, associated with active amino acid consumption and a specific feature concerning both an elevated citrate secretion and a high glutamine consumption/low glutamate secretion.

However, compared with S-ASC, V-ASC metabolism supports a stronger Warburg-like effect and a lower TCA cycle activity. This imbalance may account for the lower ability of V-ASC to differentiate into adipocytes and their inability to give rise to osteoblasts compared with S-ASC (Figure 2). Indeed, both differentiation pathways require an increased mitochondrial activity [41] and, for osteogenesis and bone formation, the accumulation of citrate and the inhibition of glycolysis [21] [42]. On the contrary, chondrogenesis, which predominantly uses the glycolytic metabolism as a source of ATP [21], was obtained from both S- and V-ASC (Figure 2).

### 3.4. Glutamine Is a Key Player in the Control of Pyruvate Routing in ASC

To better characterize the S- and V-ASC metabolic differences, cells were cultivated in partially depleted culture media.

The Warburg-like effect and the citrate production involve, respectively, the glycolysis pathway and mitochondrial TCA cycle. To decipher the respective contributions of glucose and glutamine as sources of carbon in ASC, we performed end-point experiments with cells cultivated for 96 h, in culture media completely or partially depleted in glucose and/or glutamine during the last 24 h. The replacement of the medium and the measurement on a short period and on cells at confluency were chosen to avoid the influence of substrate depletion in other metabolites than glutamine and glucose. As expected, partial or total depletion of glutamine and/or glucose affected the final number of cells. We made the choice to present metabolite concentrations without normalization on cell number because they reflected the loss of cells and do not represent what happened before. Complete results are available in Table S2. Because pyruvate is the end product of glycolysis and a substrate for the TCA cycle, these experiments were performed in pyruvate-free medium to evaluate the respective contributions of glucose and glutamine to pyruvate secretions.

**Figure 6:**
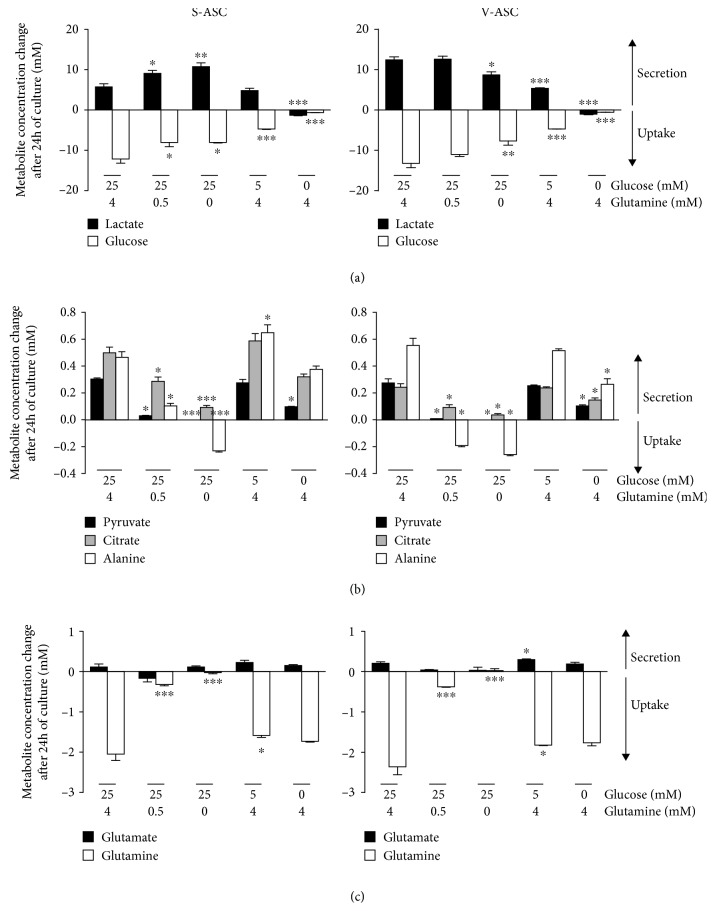
Role of glutamine and glucose in the secretome of ASC. ASC were cultivated for 72 h in fresh culture medium containing pyruvate to reach confluency. For additional 24 h of culture, the medium was replaced by pyruvate-free medium adapted to metabolomics analyses and supplemented with 0, 5, or 25 mM glucose in the presence of 0, 0.5, or 4 mM glutamine as indicated in the figure. Concentrations in culture supernatants were measured by ^1^H-NMR. Results (mean ± SEM; *n* = 3) are the difference between concentrations in cell culture supernatants and the concentration in the initial medium placed in the same conditions (control). Values were not normalized to the cell number. Negative values represent metabolite consumptions and positive values the secretions at concentrations above the control. Results for S-ASC and V-ASC are represented back to back in the figures. (a) The glycolysis pathway is evidenced by monitoring glucose uptake and lactate secretion. (b) Pyruvate, citrate, and alanine secretions and uptakes. (c) The glutaminolysis pathway is evidenced by monitoring glutamine uptake and glutamate secretion. Statistics are from the one-way ANOVA test followed by Tukey's multiple comparison test: ^∗^ *p* < 0.05, ^∗∗^ *p* < 0.01, and ^∗∗∗^ *p* < 0.001 and comparison of the partially depleted medium conditions with the complete medium condition (25 mM glucose and 4 mM glutamine). In (b) and (c), values for pyruvate and glutamate concentrations in glutamine-free conditions are represented but too low to be visible at the scale of these figures.

For both S- and V-ASC, the secretion of lactate was dependent on glucose availability (Figure 6(a) , left and right panels, respectively ). Indeed, in the absence of glucose, no lactate was secreted in the culture supernatants. On the contrary, in the absence of glucose, cells consumed the little amount of lactate initially present in the medium. Importantly, glutamine privation completely switched the metabolism of S-ASC, but not V-ASC, towards lactate synthesis. Indeed, in glutamine-free medium, lactate secretion by S-ASC increased from 6.0 ± 0.7 mM to 11.0 ± 1 mM, but glucose uptake decreased from 12.1 ± 1.1 mM to 8.0 ± 0.15 mM (Figure 6(a). This could reflect a more efficient conversion of glucose into lactate by S-ASC in the absence of glutamine, probably at the expense of the alternative uses of glucose molecules, and indicated that glycolysis in S-ASC, but not in V-ASC, was sensitive to glutamine availability.

Modulating glutamine and/or glucose supply in the culture media without pyruvate revealed that the release of pyruvate, citrate, and alanine was dose-dependently correlated with glutamine concentration but not with glucose concentrations in S- and V-ASC. Only complete glucose deprivation decreased pyruvate secretion but to a lesser extent than in the absence of glutamine (Figure 6(b)). Pyruvate is an intermediate metabolite of many pathways. It is the end product of glycolysis that can be converted into alanine by transamination and into lactate through lactate synthesis or can enter the mitochondrial tricarboxylic acid cycle to produce citrate. The partial (V-ASC) or total (S-ASC) independence of citrate and alanine secretions regarding glycolysis (Figure 6(b)) further underlines the unexpected contribution of glutaminolysis for glucose consumption. The lack of pyruvate release in the absence of glutamine indicated an increase of pyruvate consumption by both ASC. Since citrate and alanine secretions were decreased in the absence of glutamine (Figure 6(b)), we can conclude that the consumed pyruvate was not used to feed the TCA cycle nor for alanine synthesis. Accordingly, in the case of S-ASC, pyruvate seemed to be redirected to lactate synthesis, raising the Warburg-like effect to levels comparable to those obtained with V-ASC in complete medium. We did not detect a clear dose response relationship between glutamate secretion and glutamine uptake, indicating that glutamate secretion in ASC is not exclusively linked to glutamine metabolism. Glucose depletion in the medium did not affect glutamate secretion nor glutamine consumption. This indicated that glycolysis was not involved in the control of glutaminolysis (Figure 6(c)).

These results indicate that glutaminolysis is central for the use of pyruvate in TCA cycle and in the synthesis of alanine in ASC. In S-ASC only, glutamine privation also leads to a drastic switch of pyruvate consumption towards lactate production, exacerbating the Warburg-like effect. Our results reveal that sensitivity to glutamine availability is a key feature discriminating both ASC populations and evidence a complex situation where glutamine could be used as an alternative source of carbon and/or where glutaminolysis could control the fate of glycolysis products. Further analyses involving fluxomics experiments and isotope labeling will be required to finely dissect the underlying mechanisms involved.

## 4. Conclusion

Altogether, our results show that ASC have a mixed metabolism based on both glycolysis and mitochondrial activity, similar to what was already reported for bone marrow mesenchymal stem cells [21]. Interestingly, we show that glutaminolysis controls pyruvate consumption for use in TCA cycle and for alanine synthesis. Importantly, glutaminolysis also prevents the use of pyruvate for lactate production in S-ASC, but not in V-ASC. The apparent lack of this regulation level in V-ASC is compatible with the preferential use of pyruvate for lactate synthesis, probably at the expense of the mitochondrial activity, in these cells. As a consequence, S- and V-ASC can be discriminated by their relative secretion of lactate and citrate, lactate being more secreted by V-ASC and citrate by S-ASC.

To our knowledge, this study is the first one to describe at the metabolic level the differences between adipose-derived stem cells isolated from distinct adipose depots. Our results raise the possibility that mechanisms controlling the flux of pyruvate towards the synthesis of either lactate in the cytoplasm or citrate in the mitochondria could be related to the reported differences between S- and V-ASC in their proliferation and differentiation potentials. In the light of our results, the correlation between the emergence of pathologies related to the metabolic syndrome and the opposite changes affecting the expansion of the subcutaneous and the visceral adipose tissues may reflect a disruption of the metabolic program supporting ASC at the expense of their original functions.

Future challenge will be to characterize the mechanisms supporting the specific metabolism of ASC in each fat depot and the conditions leading to their breaking.

## Figures and Tables

**Table 1:** Forward and reverse sequences of primers used in RT-qPCR.

Gene	Forward sequence (5′ → 3′)	Reverse sequence (5′ → 3′)
Housekeeping		
*Rs17*	AGCCCTAGATCAGGAGATCA	CTGGTGACCTGAAGGTTAG
Adipogenic		
*Pparγ*	TCTCTCCGTAATGGAAGACC	GCATTATGAGACATCCCCAC
*Dgat2*	TGGGTCCAGAAGAAGTTCCAGAAGTA	ACCTCAGTCTCTGGAAGGCCAAAT
*Hsl*	GTGTGTCAGTGCCTATTCAG	GTCAGCTTCTTCAAGGTATC
Osteogenic		
*Dmp1*	CCCACGAACAGTGAGTCATC	CATCTCCACTGTCGTCTTCA
*Gdf15*	CGGATACTCAGTCCAGAGGT	TCGGTGCACGCGGTAGGCTT
Other		
*Ucp2*	CCTGAAAGCCAACCTCATGAC	AGGATCCCAAGCGGAGAAAGG

## Data Availability

The data used to support the findings of this study are included within the article. For each metabolomic experiment presented in this article, the supplementary tables list the results obtained for all the metabolites identified by RMN analysis, even those that were not presented in the figures.

## Supplementary Materials

Supplementary 1 Figure S1: kinetic study of the exometabolome of S-ASC. S-ASC were cultivated in medium containing pyruvate for 24 h to reach 70-80% confluency before replacement with medium of the same composition. Culture supernatants were collected after 24, 48, or 72 additional hours of culture without medium change. Cells were counted after collection of the supernatants. Concentrations have not been normalized to the cell number. (A) Kinetic of glutamine and glutamate variations. (B) Glycolysis kinetic. (C) Kinetic of citrate secretion. (D) Kinetic of the branched amino acid consumption. (E) Kinetic of alanine. (*n* = 3). (F) Cell count per well. All results are mean ± SEM.

Supplementary 2 Table S1: concentrations of metabolites measured by ^1^H-NMR in S- and V-ASC supernatants after 72 h of culture in the conditions described in Figure 5. Metabolite concentrations in the medium placed in the same culture conditions but without cells have been deduced in the presented results. Data have not been normalized. The number of cells in each well is indicated. Concentrations in S- and in V-ASC were compared. Statistics are from *t*-tests; *p* value ≤ 0.05 is considered significant.

Supplementary 3 Table S2: concentrations of metabolites measured by ^1^H-NMR in S- and V-ASC supernatants after 24 h of culture at confluency with variable concentrations of glutamine and glucose in the conditions described in Figure 6. Metabolite concentrations in the medium placed in the same culture conditions but without cells have been deduced in the presented results. Data have not been normalized. The number of cells in each well is indicated. Statistics are from one-way ANOVA followed by multiple comparison Tukey's tests. Comparisons are done relatively to the control medium culture condition (25 mM glucose and 4 mM glutamine). *p* value ≤ 0.05 is considered significant; ns: not significant.
