# Supplementary figures and images for: Metabolic Phenotyping of Adipose-Derived Stem Cells Reveals a Unique Signature and Intrinsic Differences between Fat Pads
Source: Stem Cells Int. 2019 May 14;2019:9323864. doi: 10.1155/2019/9323864 (PMC6541987; doi:10.1155/2019/9323864)

**Figure S1**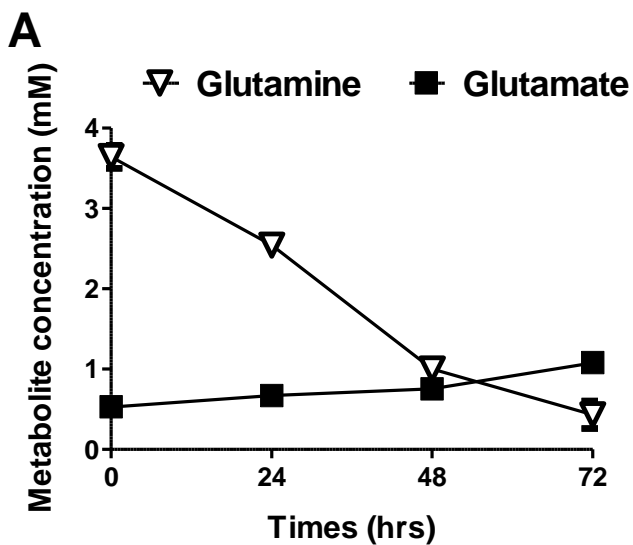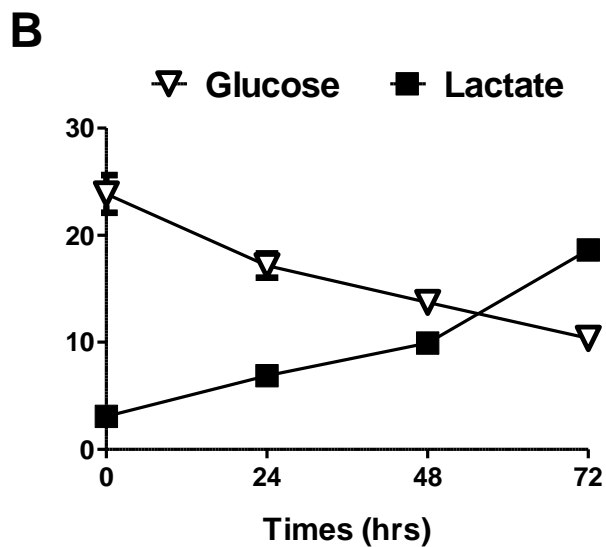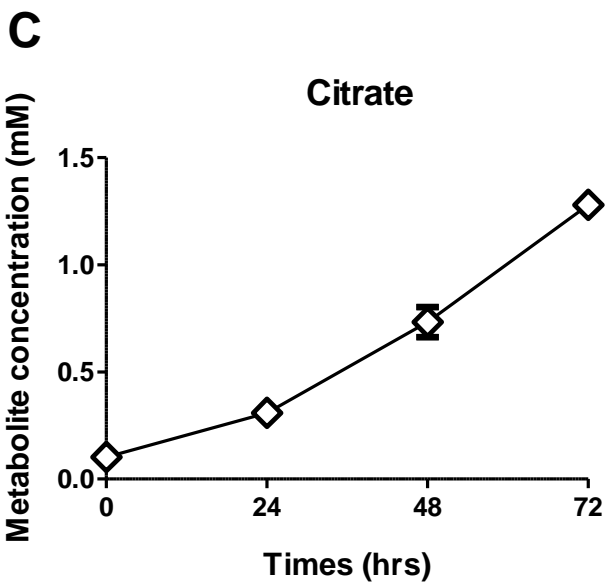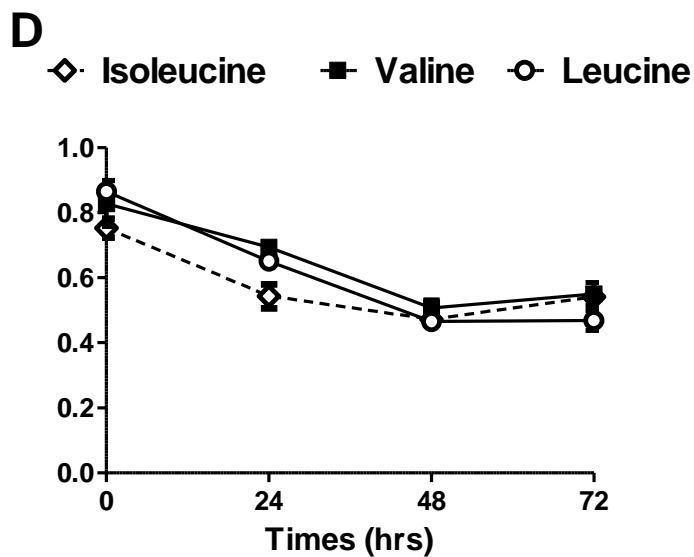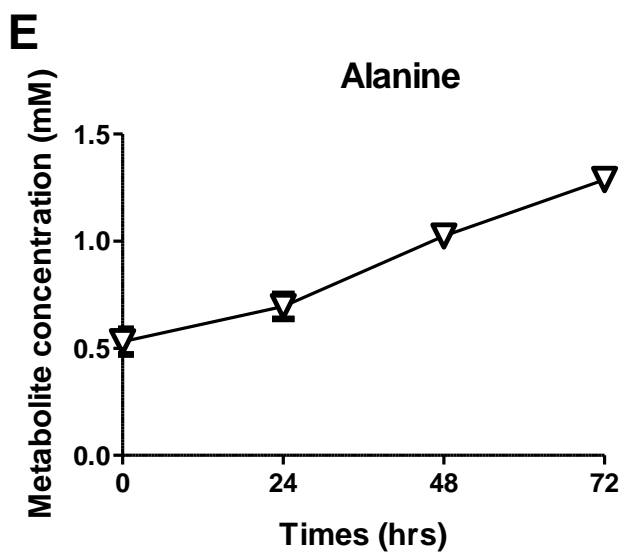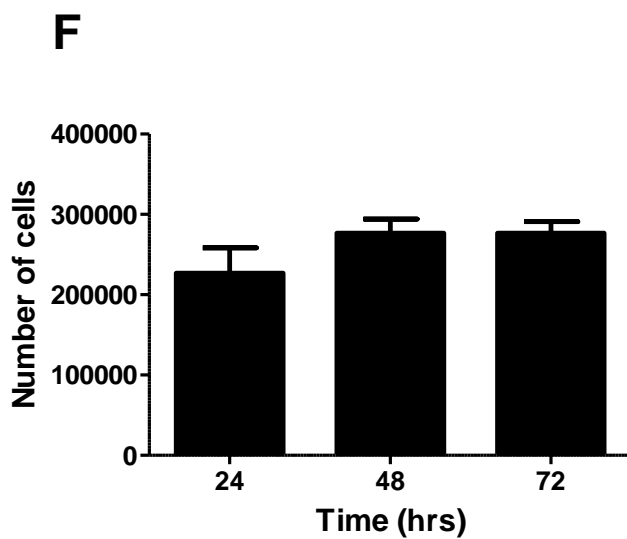

Supplement: Supplementary 1 — Figure S1: kinetic study of the exometabolome of S-ASC. S-ASC were cultivated in medium containing pyruvate for 24 h to reach 70-80% confluency before replacement with medium of the same composition. Culture supernatants were collected after 24, 48, or 72 additional hours of culture without medium change. Cells were counted after collection of the supernatants. Concentrations have not been normalized to the cell number. (A) Kinetic of glutamine and glutamate variations. (B) Glycolysis kinetic. (C) Kinetic of citrate secretion. (D) Kinetic of the branched amino acid consumption. (E) Kinetic of alanine. (n = 3). (F) Cell count per well. All results are mean ± SEM. [file 9323864.f1.pdf]
