# Supplementary material for: Metabolic Phenotyping of Adipose-Derived Stem Cells Reveals a Unique Signature and Intrinsic Differences between Fat Pads
Source: Stem Cells Int. 2019 May 14;2019:9323864. doi: 10.1155/2019/9323864 (PMC6541987; doi:10.1155/2019/9323864)
Supplement: Supplementary 2 — Table S1: concentrations of metabolites measured by 1H-NMR in S- and V-ASC supernatants after 72 h of culture in the conditions described in Figure 5. Metabolite concentrations in the medium placed in the same culture conditions but without cells have been deduced in the presented results. Data have not been normalized. The number of cells in each well is indicated. Concentrations in S- and in V-ASC were compared. Statistics are from t-tests; p value ≤ 0.05 is considered significant. [file 9323864.f2.pdf]

Concentration (in mM)	Cell number	2-Hydroxybutyrate	2-Oxoglutarate	3-Hydroxybutyrate	Acetate	Alanine	Arginine	Citrate	Formate
**SAT ASC # 1**	240 000	-0,027	0,061	0,151	-0,050	1,148	-0,374	1,951	0,334
**SAT ASC # 2**	260 000	-0,240	0,129	0,126	-0,110	0,982	-0,210	1,594	0,309
**SAT ASC # 3**	180 000	-0,008	0,132	0,103	-0,034	1,669	-0,342	2,168	0,383
**SAT ASC # 4**	200 000	-0,018	0,094	-0,066	-0,066	1,049	-0,603	1,798	0,317
**SAT ASC # 5**	180 000	-0,013	-0,088	0,050	-0,144	0,820	0,333	1,589	0,246
**SAT ASC # 6**	200 000	0,003	-0,089	0,167	-0,034	1,034	-0,085	1,780	0,303
**VAT ASC # 1**	140 000	0,029	-0,089	0,044	-0,081	1,074	0,090	1,391	0,468
**VAT ASC # 2**	200 000	-0,005	-0,070	0,255	-0,258	0,674	-0,048	0,929	0,350
**VAT ASC # 3**	140 000	0,012	-0,107	0,176	-0,199	0,734	-0,556	1,136	0,363
**VAT ASC # 4**	260 000	-0,003	-0,102	0,135	-0,281	0,732	-0,153	0,965	0,312
**VAT ASC # 5**	250 000	0,285	-0,076	0,072	0,103	0,608	-0,658	0,819	0,379
**VAT ASC # 6**	290 000	-0,008	-0,017	0,173	0,105	0,921	-0,272	0,870	0,406
**p value**	0,9116	0,1223	**0,0243**	0,278	0,7023	**0,0400**	0,7714	**<0,001**	**0,0468**

Concentration (in mM)	Fructose	Fumarate	Glucose	Glutamate	Glutamine	Glycine	Histidine	Isoleucine	Lactate	Leucine	Lysine
**SAT ASC # 1**	-0,368	0,007	-7,415	0,508	-1,689	0,412	-0,071	-0,171	19,395	-0,552	0,291
**SAT ASC # 2**	0,002	0,007	-11,124	0,493	-1,721	0,456	-0,102	-0,349	18,259	-1,039	-0,304
**SAT ASC # 3**	0,525	0,008	-7,503	0,513	-2,824	0,751	-0,118	-0,254	22,050	-0,544	0,164
**SAT ASC # 4**	0,150	0,009	-10,068	0,510	-2,749	0,433	-0,084	-0,395	19,972	-0,494	-0,117
**SAT ASC # 5**	0,060	0,006	-10,468	0,533	-1,905	0,289	-0,113	-0,452	17,635	-0,535	0,080
**SAT ASC # 6**	-0,031	0,009	-6,752	0,535	-1,478	0,782	-0,056	-0,083	18,462	-0,337	0,586
**VAT ASC # 1**	0,075	0,011	-22,501	0,568	-2,744	0,460	-0,150	-0,508	45,844	-0,695	-0,006
**VAT ASC # 2**	-0,162	0,007	-21,472	-0,033	-2,878	0,202	-0,152	-0,806	35,348	-0,857	0,018
**VAT ASC # 3**	-0,131	0,007	-24,205	0,238	-2,804	0,236	-0,159	-0,657	39,500	-0,795	0,163
**VAT ASC # 4**	-0,047	0,008	-22,156	0,142	-2,816	0,207	-0,137	-0,800	33,138	-0,846	-0,255
**VAT ASC # 5**	-0,078	0,002	-21,953	0,101	-2,731	0,170	-0,182	-0,667	32,049	-0,921	-0,229
**VAT ASC # 6**	0,048	0,007	-19,306	0,232	-2,743	0,316	-0,123	-0,562	35,286	-0,838	-0,142
**p value**	0,4162	0,6145	**<0,0001**	**0,0041**	**0,0122**	**0,0201**	**0,0009**	**0,0005**	**<0,0001**	**0,0386**	0,2105

Concentration (in mM)	Malate	Methionine	Niacinamide	Phenylalanine	Pyroglutamate	Pyruvate	Serine	Succinate	Threonine	Tryptophan	Tyrosine	Valine
**SAT ASC # 1**	0,177	0,084	-0,029	-0,009	-0,167	-0,075	-1,136	0,013	-0,461	-0,035	-0,087	-0,287
**SAT ASC # 2**	0,084	0,019	-0,029	-0,077	-0,252	-0,107	-0,731	-0,021	-0,402	-0,036	-0,050	-0,412
**SAT ASC # 3**	-0,158	0,158	-0,032	-0,098	-0,208	-0,013	-0,299	0,032	-0,230	-0,035	0,019	-0,047
**SAT ASC # 4**	0,187	0,055	-0,026	-0,006	-0,211	-0,069	-0,614	0,007	-0,318	-0,035	-0,113	-0,245
**SAT ASC # 5**	0,221	0,045	-0,030	-0,067	-0,029	-0,121	-0,596	-0,047	-0,535	-0,033	-0,134	-0,400
**SAT ASC # 6**	0,289	0,107	-0,021	0,027	0,004	-0,098	-0,637	0,030	-0,135	-0,033	-0,001	-0,200
**VAT ASC # 1**	0,140	-0,017	-0,035	-0,155	-0,168	-0,543	-0,902	0,051	-0,179	-0,047	-0,107	-0,212
**VAT ASC # 2**	-0,212	-0,105	-0,033	-0,079	-0,173	-0,547	-0,911	-0,004	0,035	-0,053	-0,142	-0,475
**VAT ASC # 3**	-0,298	-0,049	-0,031	-0,053	-0,186	-0,508	-1,044	0,015	1,312	-0,052	-0,144	-0,457
**VAT ASC # 4**	-0,087	-0,138	-0,035	-0,067	-0,184	-0,457	-1,051	-0,035	1,320	-0,042	-0,165	-0,572
**VAT ASC # 5**	-0,284	-0,083	-0,036	-0,188	-0,219	-0,573	-1,069	-0,016	1,187	-0,060	-0,224	-0,685
**VAT ASC # 6**	-0,075	-0,079	-0,031	-0,049	-0,180	-0,472	-1,059	0,028	-0,035	-0,043	-0,085	-0,650
**p value**	**0,0161**	**0,0002**	**0,0107**	0,0824	**0,0151**	**< 0,0001**	**0,0151**	0,8206	**0,011**	**0,0004**	**0,0257**	**0,0214**
