# Supplementary material for: Metabolic Phenotyping of Adipose-Derived Stem Cells Reveals a Unique Signature and Intrinsic Differences between Fat Pads
Source: Stem Cells Int. 2019 May 14;2019:9323864. doi: 10.1155/2019/9323864 (PMC6541987; doi:10.1155/2019/9323864)
Supplement: Supplementary 3 — Table S2: concentrations of metabolites measured by 1H-NMR in S- and V-ASC supernatants after 24 h of culture at confluency with variable concentrations of glutamine and glucose in the conditions described in Figure 6. Metabolite concentrations in the medium placed in the same culture conditions but without cells have been deduced in the presented results. Data have not been normalized. The number of cells in each well is indicated. Statistics are from one-way ANOVA followed by multiple comparison Tukey's tests. Comparisons are done relatively to the control medium culture condition (25 mM glucose and 4 mM glutamine). p value ≤ 0.05 is considered significant; ns: not significant. [file 9323864.f3.pdf]

S-ASC

Medium culture composition	Sample	Cell number	2-Hydroxybutyrate	2-Hydroxyisobutyrate	2-Oxoglutarate	Acetate	Alanine	Arginine	Citrate	Formate	Fumarate	Glucose	Glutamate	Glutamine	Glycine	Histidine	Isoleucine	Lactate	Leucine	Lysine	Methionine	Ornithine	Phenylalanine	Pyroglutamate	Pyruvate	Serine	Succinate	Threonine	Tyrosine	Valine
Control medium :	SATASC # 1	380 000	-0,018	-0,003	-0,180	0,041	0,545	-0,685	0,578	0,106	0,002	-10,136	0,222	-1,679	0,070	-0,054	-0,284	7,177	-0,276	-1,518	-0,089	0,643	-0,044	-0,037	0,323	-0,493	-0,021	-0,068	-0,061	-0,303
25 mM glucose	SAT ASC # 2	380 000	-0,040	0,006	-0,025	0,001	0,443	-0,585	0,474	0,094	0,002	-12,426	0,094	-2,153	-0,039	-0,075	-0,335	4,730	-0,353	-1,811	-0,129	0,480	-0,096	0,037	0,295	-0,268	-0,045	-0,156	-0,099	-0,325
4 mM glutamine	SAT ASC # 3	380 000	-0,025	-0,005	-0,222	-0,004	0,392	-0,687	0,435	0,075	0,001	-13,732	0,015	-2,268	-0,042	-0,067	-0,343	5,381	-0,258	-1,539	-0,082	1,240	-0,086	0,237	0,283	-0,484	-0,089	-0,164	-0,122	-0,414
Medium culture composition	Sample	Cell number	2-Hydroxybutyrate	2-Hydroxyisobutyrate	2-Oxoglutarate	Acetate	Alanine	Arginine	Citrate	Formate	Fumarate	Glucose	Glutamate	Glutamine	Glycine	Histidine	Isoleucine	Lactate	Leucine	Lysine	Methionine	Ornithine	Phenylalanine	Pyroglutamate	Pyruvate	Serine	Succinate	Threonine	Tyrosine	Valine
25 mM glucose	SATASC # 1	380 000	-0,008	0,000	-0,106	0,051	0,111	-0,901	0,341	0,152	0,003	-5,925	-0,005	-0,321	0,232	-0,007	-0,238	9,938	-0,143	0,773	-0,189	0,912	-0,022	0,175	0,029	-0,369	-0,040	-0,007	0,032	-0,199
0,5 mM glutamine	SAT ASC # 2	340 000	-0,012	0,000	-0,114	0,044	0,124	-0,735	0,277	0,133	0,003	-7,978	-0,339	-0,354	0,167	-0,005	-0,226	9,519	-0,214	0,002	-0,116	1,896	0,005	0,151	0,028	-0,372	0,006	0,050	0,005	-0,227
	SAT ASC # 3	400 000	-0,034	0,000	-0,115	-0,013	0,067	-1,099	0,228	0,107	0,004	-9,970	-0,150	-0,313	0,132	-0,014	-0,297	7,520	-0,233	-0,306	-0,198	1,232	-0,047	-0,172	0,021	-0,253	-0,019	-0,008	-0,048	-0,315
p value vs control		ns	ns	ns	ns	ns	<0,001	ns	<0,01	ns	<0,05	<0,05	<0,05	<0,001	<0,05	<0,001	ns	<0,05	ns	p<0,01	ns	ns	ns	ns	<0,001	ns	ns	ns	<0,05	ns
Medium culture composition	Sample	Cell number	2-Hydroxybutyrate	2-Hydroxyisobutyrate	2-Oxoglutarate	Acetate	Alanine	Arginine	Citrate	Formate	Fumarate	Glucose	Glutamate	Glutamine	Glycine	Histidine	Isoleucine	Lactate	Leucine	Lysine	Methionine	Ornithine	Phenylalanine	Pyroglutamate	Pyruvate	Serine	Succinate	Threonine	Tyrosine	Valine
25 mM glucose	SATASC # 1	400 000	0,009	0,000	-0,003	0,209	-0,216	-0,439	0,084	0,119	-0,002	-7,677	0,095	0,043	0,181	-0,013	-0,316	10,231	-0,206	0,017	-0,132	1,477	-0,009	-0,026	0,000	-0,234	-0,011	0,121	-0,020	-0,224
0 mM glutamine	SAT ASC # 2	180 000	-0,004	0,000	-0,014	0,198	-0,242	-0,515	0,069	0,121	0,000	-8,196	0,063	-0,072	0,155	-0,028	-0,359	9,247	-0,242	-0,702	-0,129	0,753	-0,099	-0,020	-0,004	-0,112	-0,021	0,040	-0,010	-0,268
	SAT ASC # 3	210 000	0,021	0,000	-0,012	0,206	-0,246	-0,770	0,118	0,144	0,000	-8,020	0,163	-0,032	0,274	0,000	-0,292	12,571	-0,129	-0,966	-0,150	1,124	0,022	-0,017	0,006	-0,344	0,008	-0,023	-0,004	-0,202
p value vs control		ns	<0,01	ns	p<0,05	p<0,001	<0,001	ns	<0,001	ns	<0,01	<0,05	ns	<0,001	<0,05	<0,01	ns	<0,01	ns	ns	ns	ns	ns	ns	<0,001	ns	ns	<0,05	<0,05	ns
Medium culture composition	Sample	Cell number	2-Hydroxybutyrate	2-Hydroxyisobutyrate	2-Oxoglutarate	Acetate	Alanine	Arginine	Citrate	Formate	Fumarate	Glucose	Glutamate	Glutamine	Glycine	Histidine	Isoleucine	Lactate	Leucine	Lysine	Methionine	Ornithine	Phenylalanine	Pyroglutamate	Pyruvate	Serine	Succinate	Threonine	Tyrosine	Valine
5 mM glucose	SATASC # 1	390 000	-0,004	0,000	-0,147	0,042	0,601	-0,325	0,529	0,132	0,004	-4,719	0,176	-1,592	0,162	-0,020	-0,173	4,578	-0,252	-0,874	-0,063	0,059	-0,116	0,000	0,274	-0,373	0,000	0,132	-0,022	-0,112
4 mM glutamine	SAT ASC # 2	280 000	-0,009	0,000	-0,155	0,026	0,579	-0,850	0,532	0,118	0,004	-4,857	0,176	-1,650	0,130	-0,035	-0,173	3,916	-0,229	-1,267	-0,125	0,294	-0,114	0,000	0,223	-0,535	-0,001	0,046	-0,028	-0,150
	SAT ASC # 3	340 000	-0,004	0,000	-0,135	0,076	0,757	-0,487	0,694	0,177	0,004	-4,799	0,318	-1,505	0,279	-0,026	-0,173	5,899	-0,135	-0,041	-0,078	0,000	-0,075	0,000	0,314	-0,207	0,044	0,159	0,031	-0,041
p value vs control		ns	ns	ns	ns	ns	<0,05	ns	ns	ns	<0,05	<0,001	ns	<0,05	<0,05	<0,01	<0,001	ns	ns	ns	ns	ns	ns	ns	ns	ns	<0,05	<0,01	<0,05	<0,01
Medium culture composition	Sample	Cell number	2-Hydroxybutyrate	2-Hydroxyisobutyrate	2-Oxoglutarate	Acetate	Alanine	Arginine	Citrate	Formate	Fumarate	Glucose	Glutamate	Glutamine	Glycine	Histidine	Isoleucine	Lactate	Leucine	Lysine	Methionine	Ornithine	Phenylalanine	Pyroglutamate	Pyruvate	Serine	Succinate	Threonine	Tyrosine	Valine
0 mM glucose	SATASC # 1	170 000	0,002	0,000	-0,172	0,026	0,356	-0,995	0,322	0,094	0,002	-0,570	0,184	-1,736	0,028	-0,024	-0,264	-1,272	-0,229	-0,402	-0,030	0,029	-0,043	-0,181	0,089	-0,213	0,001	-0,174	-0,059	-0,214
4 mM glutamine	SAT ASC # 2	120 000	0,003	0,000	-0,143	0,007	0,334	-1,646	0,282	0,106	0,002	-0,550	0,128	-1,707	0,109	-0,048	-0,264	-1,195	-0,205	-0,561	-0,062	-0,235	-0,026	-0,181	0,095	-0,532	0,009	-0,089	-0,048	-0,109
	SAT ASC # 3	270 000	0,008	0,000	-0,115	0,004	0,423	-0,539	0,347	0,113	0,003	-0,543	0,103	-1,707	0,130	-0,024	-0,264	-1,149	-0,217	-0,381	-0,025	0,059	-0,067	-0,079	0,089	-0,532	-0,005	-0,065	-0,044	-0,156
p value vs control		<0,05	<0,01	ns	ns	ns	ns	ns	<0,05	ns	ns	<0,001	ns	ns	ns	<0,05	ns	<0,001	ns	ns	ns	ns	ns	ns	<0,001	ns	ns	ns	ns	<0,05

V-ASC

Medium culture composition	Sample	Cell number	2-Hydroxybutyrate	2-Hydroxyisobutyrate	2-Oxoglutarate	Acetate	Alanine	Arginine	Citrate	Formate	Fumarate	Glucose	Glutamate	Glutamine	Glycine	Histidine	Isoleucine	Lactate	Leucine	Lysine	Methionine	Ornithine	Phenylalanine	Pyroglutamate	Pyruvate	Serine	Succinate	Threonine	Tyrosine	Valine
Control medium:	VAT ASC # 1	180 000	0,023	-0,021	-0,045	0,016	0,638	-0,950	0,231	0,110	0,001	-11,785	0,162	-2,183	-0,044	-0,080	-0,227	12,427	-0,257	-1,368	-0,183	1,998	-0,030	0,263	0,287	-0,419	-0,066	-0,147	-0,088	-0,309
25 mM glucose	VAT ASC # 2	170 000	-0,027	-0,021	-0,139	0,001	0,448	-0,679	0,206		0,003	-15,438	0,132	-2,732	-0,069	-0,088	-0,312	11,060	-0,346	-2,648	-0,204	1,213	-0,127	-0,184	0,208	-0,664	-0,042	-0,199	-0,150	-0,369
4 mM glutamine	VAT ASC # 3	140 000	0,006	-0,021	-0,251	0,034	0,560	-0,835	0,287	0,125	0,004	-12,357	0,274	-2,188	0,007	-0,065	-0,242	13,488	-0,288	-1,645	-0,115	1,360	-0,031	-0,088	0,311	-0,664	0,006	-0,046	-0,068	-0,172
Medium culture composition	Sample	Cell number	2-Hydroxybutyrate	2-Hydroxyisobutyrate	2-Oxoglutarate	Acetate	Alanine	Arginine	Citrate	Formate	Fumarate	Glucose	Glutamate	Glutamine	Glycine	Histidine	Isoleucine	Lactate	Leucine	Lysine	Methionine	Ornithine	Phenylalanine	Pyroglutamate	Pyruvate	Serine	Succinate	Threonine	Tyrosine	Valine
25 mM glucose	VAT ASC # 1	90 000	-0,001	0,000	-0,088	0,128	-0,179	-0,821	0,120	0,121	0,001	-10,337	0,006	-0,407	0,128	-0,003	-0,238	14,438	-0,286	-0,872	-0,179	2,059	-0,018	0,007	0,004	-0,369	-0,001	-0,015	-0,019	-0,289
0,5 mM glutamine	VAT ASC # 2	100 000	-0,011	0,000	-0,097	0,069	-0,179	-0,401	0,089	0,116	0,002	-10,240	0,053	-0,350	0,087	-0,035	-0,238	11,061	-0,286	-0,887	-0,179	1,912	-0,025	0,045	0,004	-0,369	-0,048	-0,105	-0,032	-0,301
	VAT ASC # 3	160 000	-0,023	0,000	-0,102	0,057	-0,211	-0,977	0,060	0,081	0,001	-12,180	-0,013	-0,391	-0,011	-0,024	-0,341	10,328	-0,273	-0,939	-0,145	2,211	-0,078	-0,068	0,004	-0,437	-0,050	-0,134	-0,072	-0,302
p value vs control		ns	ns	<0,001	ns	ns	<0,001	ns	<0,001	ns	ns	ns	ns	<0,001	ns	<0,01	ns	ns	ns	ns	ns	ns	ns	ns	<0,001	ns	ns	ns	ns	ns
Medium culture composition	Sample	Cell number	2-Hydroxybutyrate	2-Hydroxyisobutyrate	2-Oxoglutarate	Acetate	Alanine	Arginine	Citrate	Formate	Fumarate	Glucose	Glutamate	Glutamine	Glycine	Histidine	Isoleucine	Lactate	Leucine	Lysine	Methionine	Ornithine	Phenylalanine	Pyroglutamate	Pyruvate	Serine	Succinate	Threonine	Tyrosine	Valine
25 mM glucose	VAT ASC # 1	120 000	0,024	0,000	-0,016	0,247	-0,247	-0,476	0,057	0,157	0,000	-6,396	0,142	0,014	0,278	-0,023	-0,273	10,069	-0,182	-0,257	-0,132	0,952	-0,082	0,033	-0,001	-0,313	-0,004	0,054	0,004	-0,208
0 mM glutamine	VAT ASC # 2	110 000	0,009	0,000	-0,016	0,208	-0,272	-0,182	0,011	0,098	-0,001	-9,511	0,071	0,099	0,143	-0,029	-0,363	8,303	-0,162	-1,246	-0,112	1,045	-0,082	0,015	-0,010	-0,300	-0,028	-0,115	-0,023	-0,267
	VAT ASC # 3	90 000	0,014	0,000	-0,011	0,169	-0,250	-0,117	0,028	0,118	0,000	-7,197	-0,176	-0,087	0,223	-0,030	-0,338	7,881	-0,242	-0,247	-0,083	0,754	0,010	0,000	-0,005	-0,204	-0,023	-0,098	0,049	-0,189
p value vs control		ns	ns	<0,001	ns	<0,001	<0,001	ns	<0,001	ns	<0,01	<0,01	ns	<0,001	<0,01	<0,01	ns	<0,05	ns	<0,05	ns	ns	ns	ns	<0,001	<0,01	ns	ns	<0,01	ns
Medium culture composition	Sample	Cell number	2-Hydroxybutyrate	2-Hydroxyisobutyrate	2-Oxoglutarate	Acetate	Alanine	Arginine	Citrate	Formate	Fumarate	Glucose	Glutamate	Glutamine	Glycine	Histidine	Isoleucine	Lactate	Leucine	Lysine	Methionine	Ornithine	Phenylalanine	Pyroglutamate	Pyruvate	Serine	Succinate	Threonine	Tyrosine	Valine
5 mM glucose	VAT ASC # 1	100 000	-0,002	0,000	-0,184	0,037	0,534	-0,493	0,221	0,128	0,003	-4,821	0,261	-1,881	0,132	-0,033	-0,173	5,191	-0,236	-0,971	-0,094	0,412	-0,114	0,000	0,249	-0,535	0,004	0,087	-0,021	-0,067
4 mM glutamine	VAT ASC # 2	110 000	0,005	0,000	-0,097	0,049	0,490	-0,814	0,228	0,127	0,003	-4,827	0,261	-1,765	0,148	-0,050	-0,173	5,239	-0,254	-0,554	-0,063	0,118	-0,100	0,000	0,256	-0,535	-0,002	0,042	-0,024	-0,085
	VAT ASC # 3	80 000	0,007	0,000	-0,142	0,057	0,512	-0,338	0,251	0,118	0,003	-4,831	0,349	-1,794	0,099	-0,032	-0,173	5,608	-0,163	-0,705	-0,047	0,441	-0,124	0,000	0,249	-0,535	0,009	0,056	-0,022	-0,106
p value vs control		<0,05	ns	<0,001	ns	ns	ns	ns	ns	ns	ns	<0,001	ns	<0,05	<0,05	<0,05	ns	<0,001	ns	<0,05	<0,05	<0,01	ns	ns	ns	ns	ns	<0,05	ns	<0,05
Medium culture composition	Sample	Cell number	2-Hydroxybutyrate	2-Hydroxyisobutyrate	2-Oxoglutarate	Acetate	Alanine	Arginine	Citrate	Formate	Fumarate	Glucose	Glutamate	Glutamine	Glycine	Histidine	Isoleucine	Lactate	Leucine	Lysine	Methionine	Ornithine	Phenylalanine	Pyroglutamate	Pyruvate	Serine	Succinate	Threonine	Tyrosine	Valine
0 mM glucose	VAT ASC # 1	90 000	-0,008	0,006	-0,118	0,039	0,312	-0,483	0,163	0,089	0,002	-0,555	0,069	-1,813	-0,020	-0,044	-0,263	-0,799	-0,248	-1,080	-0,028	0,056	-0,056	-0,122	0,104	-0,329	0,061	-0,071	-0,068	-0,082
4 mM glutamine	VAT ASC # 2	80 000	0,007	0,000	-0,132	-0,009	0,178	-1,347	0,118	0,066	0,001	-0,555	0,168	-1,852	-0,092	-0,046	-0,264	-1,319	-0,293	-0,699	-0,119	0,029	-0,093	-0,145	0,076	-0,532	0,006	-0,200	-0,105	-0,302
	VAT ASC # 3	110 000	0,017	0,000	-0,074	0,046	0,289	-1,239	0,159	0,098	0,002	-0,557	0,264	-1,621	0,028	-0,019	-0,264	-0,737	-0,151	-0,635	-0,036	-0,353	-0,029	-0,093	0,115	-0,451	0,028	-0,089	-0,054	-0,193
p value vs control		<0,05	ns	<0,001	ns	ns	<0,001	ns	<0,05	ns	ns	<0,001	ns	<0,01	ns	<0,05	ns	<0,001	ns	ns	<0,05	<0,001	ns	ns	<0,001	ns	ns	ns	ns	ns
